# Coupling cognitive and brainstem dysfunction in multiple sclerosis-related chronic neuropathic limb pain

**DOI:** 10.1093/braincomms/fcac124

**Published:** 2022-05-17

**Authors:** Peter Foley, Yazhuo Kong, Ramune Dirvanskiene, Maria valdes-Hernandez, Matteo Bastiani, Jonathan Murnane, Robin Sellar, Neil Roberts, Cyril Pernet, Christopher Weir, Thomas Bak, Lesley Colvin, Siddharthan Chandran, Marie Fallon, Irene Tracey

**Affiliations:** 1 Anne Rowling Regenerative Neurology Clinic, University of Edinburgh, 49 Little France Crescent, Edinburgh EH16 4SB, UK; 2 CAS Key Laboratory of Behavioural Science, Institute of Psychology, Chinese Academy of Sciences, Beijing, China; 3 Department of Psychology, University of Chinese Academy of Sciences, Beijing, China; 4 Dementia Research Institute, University of Edinburgh, Edinburgh, UK; 5 Centre for Clinical Brain Sciences, University of Edinburgh, Edinburgh, UK; 6 Sir Peter Mansfield Imaging Centre, School of Medicine, University of Nottingham, Nottingham, UK; 7 NIHR Biomedical Research Centre, University of Nottingham, Nottingham, UK; 8 Wellcome Centre for Integrative Neuroimaging, University of Oxford, Oxford, UK; 9 Clinical Research Imaging Centre, Edinburgh University, Edinburgh, UK; 10 Edinburgh Clinical Trials Unit, Usher Institute, University of Edinburgh, Edinburgh, UK; 11 School of Philosophy, Psychology and Language Sciences, University of Edinburgh, Edinburgh, UK; 12 Division of Population Health and Genomics, University of Dundee, Dundee, UK; 13 Department of Palliative Medicine, University of Edinburgh, Edinburgh, UK; 14 Nuffield Department of Clinical Neurosciences, John Radcliffe Hospital, University of Oxford, Oxford, UK

**Keywords:** pain, neuropathic, multiple sclerosis, cognition, brainstem

## Abstract

Chronic pain in multiple sclerosis is common and difficult to treat. Its mechanisms remain incompletely understood. Dysfunction of the descending pain modulatory system is known to contribute to human chronic pain conditions. However, it is not clear how alterations in executive function influence this network, despite healthy volunteer studies linking function of the descending pain modulatory system, to cognition. In adults with multiple sclerosis-associated chronic neuropathic limb pain, compared to those without pain, we hypothesized altered functional connectivity of the descending pain modulatory system, coupled to executive dysfunction. Specifically we hypothesized reduced mental flexibility, because of potential importance in stimulus reappraisal. To investigate these hypotheses, we conducted a case-control cross-sectional study of 47 adults with relapsing remitting multiple sclerosis (31 with chronic neuropathic limb pain, 16 without pain), employing clinical, neuropsychological, structural, and functional MRI measures. We measured brain lesions and atrophy affecting descending pain modulatory system structures. Both cognitive and affective dysfunctions were confirmed in the chronic neuropathic limb pain group, including reduced mental flexibility (Delis Kaplan Executive Function System card sorting tests *P* < 0.001). Functional connectivity of rostral anterior cingulate and ventrolateral periaqueductal gray, key structures of the descending pain modulatory system, was significantly lower in the group experiencing chronic neuropathic pain. There was no significant between-group difference in whole-brain grey matter or lesion volumes, nor lesion volume affecting white matter tracts between rostral anterior cingulate and periaqueductal gray. Brainstem-specific lesion volume was higher in the chronic neuropathic limb pain group (*P* = 0.0017). Differential functional connectivity remained after correction for brainstem-specific lesion volume. Gabapentinoid medications were more frequently used in the chronic pain group. We describe executive dysfunction in people with multiple sclerosis affected by chronic neuropathic pain, along with functional and structural MRI evidence compatible with dysfunction of the descending pain modulatory system. These findings extend understanding of close inter-relationships between cognition, function of the descending pain modulatory system, and chronic pain, both in multiple sclerosis and more generally in human chronic pain conditions. These findings could support application of pharmacological and cognitive interventions in chronic neuropathic pain associated with multiple sclerosis.

## Graphical Abstract

**Graphical Abstract:**
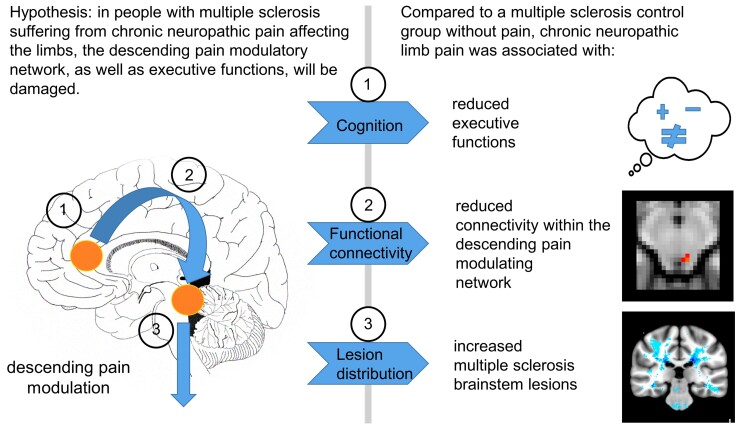

## Teaser

Foley *et al*. report evidence of dysfunction of descending pain modulation, in adults with multiple sclerosis experiencing chronic neuropathic pain. In comparison to multiple sclerosis controls, they identify (i) reduced functional connectivity of rostral anterior cingulate cortex and brainstem periaqueductal gray, (ii) executive dysfunction, and (iii) higher brainstem lesion volume.

## Introduction

Multiple sclerosis is a chronic neuroinflammatory and neurodegenerative disorder that is an important cause of neurological disability in adults.^1^ Chronic pain frequently affects people with multiple sclerosis (pwMS) at all disease stages, as does cognitive dysfunction.^2^ Among the various pain syndromes which can affect pwMS, chronic neuropathic limb pain (cNLP), a specific pain syndrome which most commonly affects the lower limbs, affects around 26% of pwMS.^3^ It is often refractory to pharmacotherapy, and mechanisms are poorly understood. Improved understanding of the mechanisms of cNLP in multiple sclerosis, including of any associated cognitive dysfunction, could aid development of pharmacological or psychological therapies.^4^

Chronic pain is a subjective multidimensional state, modulated by behavioural, affective, and cognitive factors. The latter include expectation, attention, and reappraisal. The descending pain modulatory system (DPMS) is a well-characterized network of cortical, subcortical and brainstem structures (including rostral anterior cingulate cortex—rACC, and periaqueductal gray—PAG), which is associated with both cognitive and affective functions.^5,6^ The ACC, along with prefrontal cortex, has been implicated in mediating cognitive and affective conditions, the former including mental flexibility and reappraisal.^7,8^ PAG has a key role in coding and relaying descending modulation via the rostral ventromedial medulla, to the dorsal horn of the spinal cord^6,9^ and additionally in attentional analgesia during cognitive demand.^10^

In preclinical models the DPMS has been demonstrated to exert top-down inhibitory and facilitatory influences on spinal dorsal horn gating of afferent nociceptive input by opioidergic, serotoninergic and noradrenergic pathways. Furthermore, preclinical chronic pain states have repeatedly been linked to an imbalance between inhibitory and facilitatory DPMS functions.^5,11^ In human volunteers the DPMS has been demonstrated to play a key role in experimentally induced placebo analgesia, and central pain sensitization.^12^

Evidence of DPMS dysfunction in chronic neuropathic pain associated with human disease is growing.^5^ Key involvement of the rACC and PAG, amongst other regions, has been repeatedly confirmed. Existing studies have highlighted in healthy volunteers how cognitive manipulations affect DPMS function.^13–15^ However, the presence of cognitive impairment alongside DPMS dysfunction associated with chronic pain is underexplored.

Clinical and behavioural data in pwMS with chronic pain could be compatible with known features of DPMS dysfunction, including affective disturbance, hypersensitivity and allodynia. The latter may reflect impaired inhibitory actions or enhanced facilitatory actions of DPMS.^16,17^ There is limited evidence however to clarify the role of multiple sclerosis related lesions or atrophy in pain syndromes affecting pwMS^18^ overall. Furthermore, to the authors’ knowledge, no study of DPMS function or structure in multiple sclerosis has included measures of lesions or atrophy. One study, employing a healthy control group, assessed the interaction between resting state functional MRI (rs-fMRI) functional connectivity (FC) networks in pwMS with mixed neuropathic pain features, and described altered interaction of DPMS networks with other FC networks.^19^ Magnetoencephalography was also used to study dynamics of resting state networks.^20,21^ The intrinsic function of DPMS, including any role of lesions or atrophy, as well as associated cognitive performance,^22^ remains to be clarified.

In pwMS affected by cNLP, we hypothesized dysfunction of DPMS linked to both cognition and affect. Cognitive components specifically include mental flexibility and affective components include depression and anxiety.^6,23,24^ We further hypothesized that multiple sclerosis-related atrophy or lesion distribution would affect DPMS structures. Therefore, we designed a cross-sectional case:control study of adults with relapsing remitting multiple sclerosis, comparing those with cNLP, to a disease control group without pain. Furthermore, we used structural and functional MRI of the brain to test for both the presence of structural abnormalities affecting known DPMS structures, and differential functional connectivity of the DPMS.

## Materials and methods

### Experimental design and recruitment

A cross-sectional case-control study was conducted. Participants with relapsing remitting multiple sclerosis, either with the specific pain syndrome of cNLP or without pain, were recruited from an outpatient clinic (www.annerowlingclinic.org) which provides specialist care to approximately 2000 pwMS in South East Scotland. We recruited pwMS experiencing cNLP specifically, in order to maximize clinical homogeneity of our sample. We recruited a control group of participants also living with RRMS because of the potential for numerous known and unknown confounders specifically associated with multiple sclerosis^1^ and with chronic disease more generally. This was judged to be a more relevant control group in comparison to using healthy controls. Participants were referred by the treating clinical team. Study groups were recruited to ensure close comparability of age and gender at the group level. Consent was obtained according to the Declaration of Helsinki. All procedures were approved by the West of Scotland Research Ethics Committee (13/WS/0094).

Inclusion criteria included definite relapsing remitting multiple sclerosis,^25^ age over 18 and fluency in English. cNLP was defined as continuous or near-continuous pain affecting one or more limbs, of at least 3 months’ duration; with existing clinical diagnosis of neuropathic pain made by multiple sclerosis or pain specialist. We preferred the term ‘chronic neuropathic limb pain’ in contrast to previously used labels including ‘central pain’, as the latter might include a number of other pain syndromes attributed to central mechanisms. A trained neurologist (P.F.) furthermore excluded alternative aetiologies of pain by history and examination and confirmed presence of neuropathic pain by research criteria.^26^ Control group participants confirmed absence of pain, including previous experience of chronic pain, at interview with a single investigator (P.F.). Furthermore, all control group participants confirmed absence of chronic pain at any time using the following standardized questionnaire based on existing literature.^27^ ‘Throughout our lives, most of us have had pain from time to time (such as minor headaches, sprains, and toothaches). Have you had pain other than these everyday kinds of pain within the last 24 h, or do you have a problem with pain which has lasted for more than 3 months?’.

Exclusion criteria included known severe cognitive deficit, presence of hemi-body pain (which is likely to arise due to different mechanisms),^18,28^ use of strong opioids,^29^ clinically confirmed MS relapse or steroid treatment within the preceding month, and psychiatric disease judged severe enough to preclude tolerance of the study protocol. To enhance generalizability of the study to clinical populations, participants with known psychiatric conditions were not otherwise excluded. Although differences in the pathobiology of pain syndromes have been reported between biological sexes,^30,31^ we recruited both female and male participants, as while incidence and prevalence of multiple sclerosis are higher in females both in the UK.^32^ and internationally,^1^ exclusion of either sex would limit generalizability of our reported findings.^31^ Participants were not required to stop medications, including analgesia. In the absence of existing comparable examination of DPMS structure and function in multiple sclerosis, sample size was based on comparable studies in clinical chronic pain states.^13–15^

### Pain severity and symptom distribution

The Brief Pain Inventory (short form)^27^ was used to derive the pain severity index (sum of ‘average’, ‘worst’ and ‘least’ pain, and ‘pain at time of assessment’). Location of pain was recorded by cNLP group participants on a standardized body map. Control group participants recorded location of painless sensory symptoms (including numbness, tingling) using an identical procedure.^27^ To generate group-level symptom distribution maps, individual symptom maps were summed, then averaged, using MatlabR2014a.

### Acquisition of clinical and symptom data

All participants underwent clinical assessment by a neurologist (P.F.) in a single session. The following were recorded in all participants: Age, sex, time since diagnosis of multiple sclerosis in years, current medication (confirmed from medical records), Expanded Disability Status Scale,^33^ Hospital Anxiety and Depression Scale (HADS),^34^ Fatigue Severity Scale,^35^ Pittsburgh Sleep Quality Inventory,^36^ and years of full-time education. In participants with cNLP, pain catastrophizing^37^ was measured. In a post-hoc analysis, in order to illustrate clinical relevance of the data on depression and anxiety symptoms ^27^ presented, the number of participants in each group meeting thresholds for major depression and generalized anxiety disorder were calculated using a threshold of eight and above for both HADS-depression, and HADS-anxiety separately, according to existing literature.^38^

Quantitative Sensory Testing (QST) tested for features compatible with central sensitization^17^ using an abbreviated procedure^39^ administered by a single investigator (P.F.). Measures included pain sensation to single pinprick (numerical rating scale 0–10, anchors at ‘no pain’ and ‘worst pain imaginable’) (Neurotips, Owen Mumford, UK). Mechanical, thermal and cold allodynia were defined by reported pain sensation in association with standardized stimuli using; calibrated brush approximately 100 mN, temperature-controlled rollers at 40 and 25 degrees Centigrade respectively (Rolltemp, Somedic, Sweden). Wind-up ratio (ratio of participant pain rating following 10 sequential pinprick stimuli administered at 1 Hz, to pain rating to single pinprick stimulus) was measured. QST was completed at least an hour before imaging in all cases, with the exception of wind-up which was tested after imaging to avoid contamination of functional imaging measures. Normative data for the QST measures employed are available from the authors at request.

### Neuropsychological assessment

All participants underwent focussed cognitive assessment in order to examine cognitive deficits commonly observed in multiple sclerosis,^40^ as well as, specifically, executive functions including mental flexibility, potentially relevant to DPMS function. Anatomical localization of functions tested by these instruments overlaps with structures involved in cognitive and affective modulation of pain, including rACC and dorsolateral prefrontal cortex.^8^ A neuropsychological battery was administered by a neurologist (P.F.) or neuropsychologist (R.D.) in a single session on the same day as clinical assessment, including Brief International Cognitive Assessment for Multiple Sclerosis (BICAMS)^40^ letter fluency (letter S) and constrained fluency (letter T, four-letter words),^41^ reverse digit span, Hayling sentence completion task and letter:number alternation,^41^ Delis-Kaplan Executive Function System card sorting tests,^42^ and the Test of Everyday Attention elevator test with distraction.^43^

### Brain imaging acquisition

Structural and rs-fMRI of the brain was acquired in a single session, preceded by administration of the Brief Pain Inventory in all cases.^27^ MRI imaging was performed on a single Siemens Verio 3 tesla system (Erlangen, Germany). A 12-channel phased array head coil (Siemens) was used. For each participant structural imaging was acquired as follows, field of view 256 × 256 × 160 mm and isotropic 1mm^3^ voxels: T1-weighted (Magnetization-prepared rapid gradient-echo, flip angle 9 degrees, TR 2300 ms, TE 2.98 ms, TI 900 ms); T2-weighted (TR 3200 ms, TE 416 ms) and FLAIR (Fluid-Attenuated Inversion Recovery, TR 5000 ms, TE 402 ms, TI 1800 ms). Phase and magnitude field maps (Siemens) were then acquired before resting state echo-planar images (TR 3000 ms, TE 30 ms, flip angle = 90 degrees, 46 slices, field of view 192 mm, 3 mm slice thickness, duration 5 min 23 s, 107 volumes). Pain severity due to multiple sclerosis (Numerical Rating Scale ranging from 0–10) was recorded immediately prior to rs-fMRI acquisition.

### Statistical analysis

All study data was identified by a computer-generated random numerical code.

### Clinical/neuropsychological data

Data were analysed in R (www.r-project.org). Statistical significance was accepted at the two-sided 5% level. Bonferroni correction for multiple comparisons was applied for each family of tests. Data distribution was assessed by histogram and Quantile–Quantile plots. Wilcoxon rank-sum test or *t*-test was used to compare continuous measures between groups. Chi-squared or Fisher exact tests were used to compare categorical variables between groups. Correlation was tested by Spearman’s Rho or Pearson’s correlation coefficient. We did not employ multivariate analyses in examining associations of pain severity within the cNLP group, because we considered that the sample size available would not allow reliable interpretation of these analyses.

### Imaging data

Analysis implemented FMRIB Software Library (FSL, v5, www.fmrib.ox.ac.uk^44^), Mango Image Viewer [v3.4, (2015)] and SPMv12 (www.fil.ion.ucl.ac.uk/spm/). Use of standard space templates allowed use of open source probabilistic atlases^45^ and comparability with fMRI data. Intracranial volume (ICV) was calculated by multispectral segmentation. All co-ordinates follow Montreal Neurosciences Institute (MNI) convention. All registrations were confirmed visually.

### Semi-automated segmentation of multiple sclerosis lesions and lesion distribution probability maps

An experienced neuroradiologist (R.S.), blinded to participant pain status, detailed multiple sclerosis lesion location for each participant, based on MPRAGE, T2 and FLAIR data, in an anonymized written report. Intensity thresholding of each participant’s FLAIR images at greater than two standard deviations above mean image intensity of the brain parenchyma was separately carried out within the ICV mask, using in-house software implemented in MatlabR2014a, to generate a provisional binary lesion map for each subject. The resultant lesion map was manually edited by a trained neurologist (P.F.), guided by the neuroradiological assessment, in axial and sagittal planes.

Each resulting lesion mask was registered to the MNI 152 1 mm standard space template using a nonlinear registration procedure implementing FSL’s FNIRT.^46^ Group-level lesion probability maps were created, using Matlab, by summing individual maps and then averaging in MNI 1 mm space. Subject-wise lesion volumes were calculated as a percentage of ICV for each subject. Lesion distribution in cNLP and control groups was compared visually by subtraction of lesion distribution masks with the contrasts of cNLP > control and control > cNLP.^47^

### Voxel-based morphometry

Voxel-based morphometry (VBM) processing followed standard published protocols,^48^ including extraction of brain data, segmentation of grey matter (GM) and registration to the MNI 152 2 mm template using non-linear registration. Modulated GM images were smoothed with an isotropic Gaussian kernel (sigma 3 mm, approximate full-width-half-maximum 7 mm). A voxel-wise general linear model (two-sample unpaired *t*-test with covariates) was applied using FSL randomize with threshold free cluster extent (TFCE) and 5000 iterations. For each subject, ICV and age were included in all VBM analyses as nuisance covariates. Lesion filling was carried out in each participant’s native T1 space.^44^ Statistical significance was accepted at the 5% level, corrected for multiple comparisons, TFCE.

A single binarized mask of key cortical and subcortical structures relevant to descending modulation of pain was employed as a region of interest (ROI) in VBM analyses. Masks of dorsolateral prefrontal cortex (DLPFC), insula, brainstem, ACC, amygdala, orbitofrontal cortex and frontal pole^8^ were summed to create a single mask of key DPMS structures. Apart from DLPFC,^49^ masks were calculated from relevant atlases, thresholded at 50% probability then binarized.^45^ We selected the cortical and subcortical structures included, based on existing literature. We did not specify subregions of cortical/subcortical structures in order to generate an inclusive mask, given that structural correlates of chronic pain in pwMS, are largely unknown.^18,19^

In addition, in order to inform interpretation of fMRI data by identifying trends in GM volume not reaching the specified threshold for statistical significance, but which might affect GM volume within the defined ROIs used for fMRI analyses, the described whole-brain VBM analyses were repeated at the exploratory threshold of *P* < 0.001, uncorrected for multiple comparisons.

### Resting state functional MRI data

Preprocessing of rs-fMRI data used standard procedures in FMRIB Expert Analysis Tool (FEAT) version 6, implemented in FSL.^44^ The following steps were taken: removal of first three volumes to allow signal equilibration,^14^ high pass filter cutoff of 90 s applied, head motion calculation, slice timing correction (using a Fourier-space time-series phase-shifting), spatial smoothing with a full-width-half-maximum kernel of 5 mm^13^ and grand-mean intensity normalization of the entire four-dimensional data set by a single multiplicative factor. Each participant’s rs-fMRI data were registered to their own brain-extracted T1 data using a linear registration in FSL’s FLIRT, including boundary based registration and field map unwarping.^44^ Resting state fMRI data were then registered to the MNI152 2 mm brain-extracted template using a non-linear transformation with 12 degrees of freedom.^46^

Participants manifesting absolute head displacement of one millimetre or more during rs-fMRI acquisition were excluded from further analysis. Median participant head movement was then calculated and compared across groups. Independent components analysis (FSL MELODIC)^44^ was used to identify artefacts and manually ‘de-noise’ each participant’s data by regressing out components of no interest including head movement, CSF signal, and white matter signal. Furthermore, six head movement parameters, along with white matter and CSF timecourses for each participant were employed as nuisance covariates in linear modelling.

ROI masks were created for the rACC and PAG. All masks were binarized spherical masks of 6 mm radius (905 mm^3^).^9^ The rACC mask was based on location of previously published rostral ACC seeds.^50^ and lay within described boundaries of the rACC,^51^ centred at x = 0, y = 42, z = 8. In common with other groups,^19^ we selected an estimate of PAG identical to a previously published estimate,^9^ centred on x = 0, y = −32, z = −10. This was identified as an inclusive estimate of whole PAG for our analysis, as the role of PAG sub-regions in pain associated with multiple sclerosis has not previously been well defined.^18,19^ A further ‘control’ mask was created to test specificity of any findings to the DPMS. This mask was centred on the midline in the occipital cortex, which has no known DPMS role (x = 0, y = 74, z = 8).

Subject-wise time courses were calculated for the rACC ROI, from data pre-processed as described. The resultant time course was used as the independent variable in a general linear model examining correlation with time courses of all other voxels in the brain, after regressing out correlation attributable to head motion, CSF or white matter signal as described above. A mixed effects model controlling for Family Wise Error with FMRIB’s Local Analysis of Mixed Effects version 1 was implemented in FEAT.^44^ A Z threshold of 2.3 and cluster forming *P* < 0.05 were used. In order to calculate FC between the rACC seed and PAG ROI, a small volume comparison was calculated within the PAG ROI using FSL’s randomize with settings as described above. The above analyses were repeated in separate post-hoc analyses, including firstly brainstem-specific multiple sclerosis lesion volume, and secondly participant sex, as covariates of no interest. Linear correlations between rACC FC to PAG, and pain severity scores at the time of imaging within the cNLP group were also calculated. We did not include additional clinical variables in the described models because of limitations in sample size.

### Examination of multiple sclerosis lesion distribution

To explore any differential distribution of multiple sclerosis lesions in the cNLP group, multiple sclerosis lesion volume specifically affecting regions of interest relevant to DPMS connectivity was calculated as a post-hoc analysis. Lesion volume falling within rACC:PAG white matter tracts, and the whole brainstem, was calculated separately for each participant.

Bilateral probabilistic masks of rACC:PAG white matter tracts were derived from diffusion MRI in 500 randomly selected minimally pre-processed participants in the Human Connectome Project.^52^ Probabilistic tractography was run using Probtrackx2.^53^ Voxel-wise raw streamline counts were converted to probabilities and averaged across participants to obtain a ‘standardized’ tract-specific spatial map in MNI152 template space. This map was then thresholded at 0.01 and binarized to obtain a tract-specific ROI. Binary masks of brainstem were adapted from the Harvard-Oxford atlas, thresholded at 50% probability.^44^

The volume of multiple sclerosis lesions overlapping with binarized masks of brainstem and of rACC:PAG tracts was then calculated for each participant in native space by warping tract and brainstem masks to each participant’s native space images using FSL’s FNIRT.^46^ Lesion volumes within each ROI were expressed as a percentage of ICV.

Additionally, to explore differential lesion distribution within the brainstem specifically, an analysis of lesion distribution was carried out within the brainstem mask as described. We used permutation testing implemented in FSL’s Randomize as described above (TFCE, *P* < 0.05 corrected). Brainstem lesion location was assigned according to a brainstem histopathological and MRI atlas.^54^

### Data availability

Anonymized data will be shared on suitable request from a qualified investigator.

## Results

### Demographic and clinical characteristics of study groups

Forty-seven adults with relapsing remitting multiple sclerosis were recruited. Thirty-one had cNLP, and 16 were without pain, as defined above. There was no significant difference in severity of disability, disease duration, or years of education nor in medications, apart from more frequent prescription of gabapentinoid medications in the cNLP group (Table 1). Approximately 80% of participants were female, comparable to sex-specific multiple sclerosis prevalence ratios in UK and globally.^1^ Participant age fell within the range expected for pwMS in the UK population.

**Table 1:** Demographics and medication

	Control (MS without pain) *n* = 16	MS with cNLP *n* = 31	Statistical test	*P* value
Gender (% female)	81.2	80.6	NA	
Age (years), median (IQR)	42.50 (33.00–52.25)	41.0 (38.0–52.0)	NA	
EDSS, median (IQR)	1.75 (1.0–2.12)	2.00 (1.50–3.02)	W	0.23
Disease duration (years), median (IQR)	7.75 (3.37–13.62)	7.50 (5.00–13.00)	W	0.73
Years full time education (years), median (IQR)	15.00 (13.00–18.50)	15.50 (12.00–17.75)	W	0.34
Pain Severity (Pain Severity Index, range 0–40), median (IQR)	NA	17.00 (10.00–22.00)	NA	NA
Pain Duration (years), median (IQR)	NA	6.00 (2.25–8.75)	NA	NA
Medications				
Weak opiates (%) (regular codeine-containing medication)	0 (0%)	3/31 (9.7%)	Fisher	0.54
Any antidepressant medication	6/16 (37.5%)	18/31 (58.1%)	χ^2^	0.30
Adjuvant analgesic: tricyclic antidepressant	3/16 (18.75%)	11/31 (35.5%)	Fisher	0.32
Adjuvant analgesic: gabapentinoid (pregabalin or gabapentin)	0/16 (0%)	17/31 (54.8%)	Fisher	0.0001 *
Baclofen	1/16 (6.25%)	4/31 (12.9%)	Fisher	0.65
MS DMT (%)	14/16 (87.5%)	19/31 (61.2%)	Fisher	0.094

*Statistically significant at 5% level. No correction for multiple comparisons applied.

EDSS, Expanded Disability Status Scale; Fisher, fisher exact test; IQR, interquartile range; MS DMT, multiple sclerosis disease modifying therapy; NA, not applicable; SD, standard deviation;

W, Wilcoxon rank sum test, χ^2^ = chi-squared test.

Complete medications are listed in the online supplement (Supplementary Table 1).

### Distribution of painful and painless neuropathic sensory symptoms

**Figure 1 Symptom distribution mapping.**
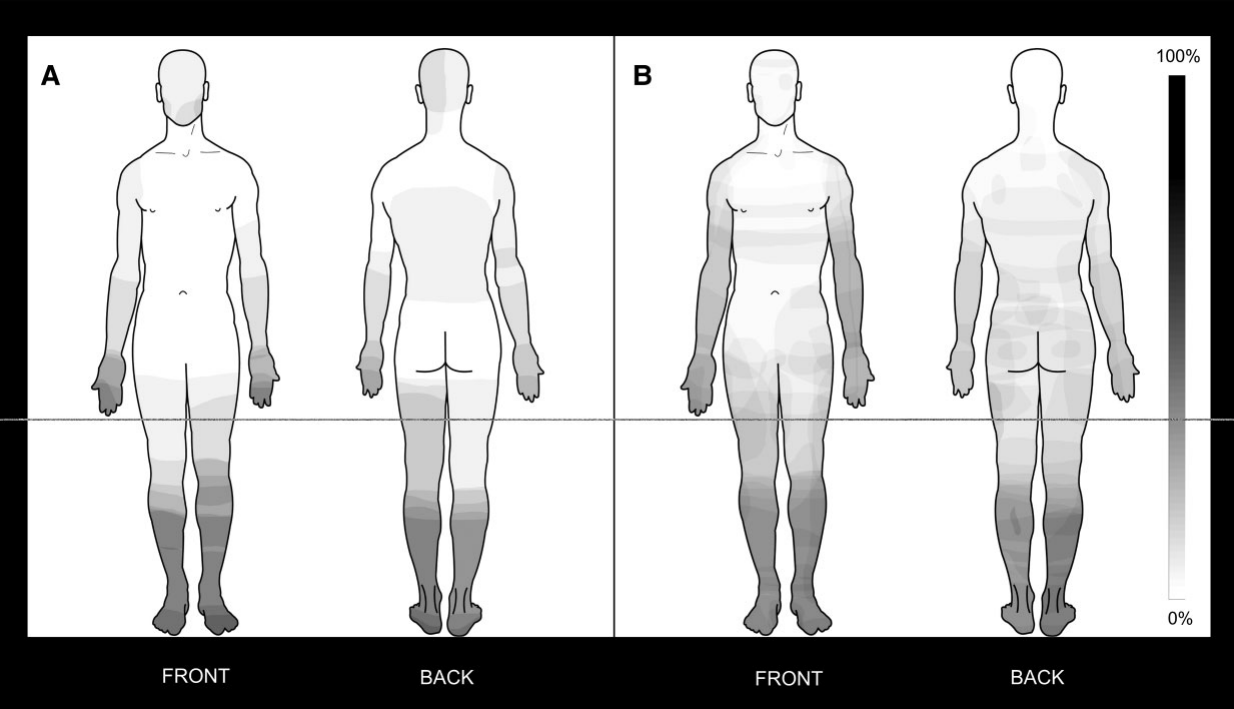
Average group distribution of painless (**A**, control group) and painful (**B**, cNLP group) neuropathic sensory symptoms. Each study participant hand-drew on a standardized body template the location of their neuropathic pain (cNLP group only, *n* = 31) or the location of their non-painful neuropathic sensory disturbance (control group only, *n* = 16). Individual symptom maps were summed within each group separately and averaged to create group-level symptom distribution maps. Heatmaps denote likelihood of symptom location in the control group (left) and cNLP group (right). Greyscale shading denotes probability of symptom report in each location at the group level (0% = symptom reported at this location by no participants in the group; 100% = symptom reported at this location by all participants in the group). Symptoms affected all four limbs in both groups, with higher symptom likelihood in the lower limbs.

Fourteen of 16 control participants (87.5%) experienced painless neuropathic sensory disturbance in the limbs. Two experienced no sensory disturbance. Distribution of neuropathic sensory symptoms (neuropathic pain in cNLP group, painless sensory disturbance in control group) was confirmed. Both groups described symptoms preferentially affecting the lower limbs (Fig. 1).

### Affective symptoms, sleep, fatigue and quantitative sensory testing

Presence of cNLP was significantly associated with symptoms of depression and fatigue (Table 2). No control group patients (0/16, 0%) met a previously published threshold for major depression.^38^ in comparison to 11 (35.5%) of the cNLP group. Seven (43.8%) of the control group participants, and 19 (61.3%) of the cNLP participants met a previously published threshold for generalized anxiety disorder.^38^

**Table 2:** Symptoms of affective disturbance and fatigue

	Control (MS without pain) *n* = 16	MS with cNLP *n* = 31	Statistical test	*P* value
Depression (HADS), median (IQR)	1.50 (0.00–6.00)	5.00 (2.00–8.50)	W	0.0051*
Anxiety (HADS), median (IQR)	5.50 (2.00–9.25)	9.00 (6.00–12.00)	W	0.0266
Fatigue (FSS)), median (IQR)	33.00 (26.75–43.75)	51.00 (43.50–56.50)	W	0.0034*
Sleep quality (PSQI)), median (IQR)	6.00 (3.75–7.25)	8.00 (5.50–12.00)	W	0.0278

*Statistical significance at 5% level after Bonferroni correction for multiple comparisons (four comparisons, threshold *P* = 0.0125).

cNLP, chronic neuropathic limb pain; FSS, Fatigue Severity Scale; HADS, Hospital Anxiety and Depression Scale; IQR, interquartile range; MS, multiple sclerosis; PSQI, Pittsburgh Sleep Quality Inventory; W, Wilcoxon rank sum test.

Allodynia was observed only in the cNLP group. No statistically significant difference was found in QST measures, after Bonferroni correction (Table 3).

**Table 3:** Quantitative sensory testing

	Control (MS without pain) (*n* = 16)	MS with cNLP (*n* = 31)	Statistical test	*P* value
Single pinprick pain rating (no pain = 0, maximum pain imaginable = 10), median (IQR)	0.00 (0.00–0.50)	1.00 (0.00–2.00)	W	0.019
Wind-up ratio greater than 1, count (percentage)	4/16 (25%)	18/31 (58.0%)	Fisher	0.065
Any allodynia (thermal or dynamic mechanical), count (percentage)	0/16 (0%)	8/31 (25.8%)	Fisher	0.038

*Statistical significance at 5% level after Bonferroni correction for multiple comparisons (three comparisons, threshold *P* = 0.0167).

cNLP = chronic neuropathic limb pain; Fisher, Fisher exact test; IQR, interquartile range; MS, multiple sclerosis; WUR, wind up ratio; W, Wilcoxon rank sum test.

### Neuropsychological assessment

cNLP was associated with lower scores for measures of mental flexibility. Performance approached ceiling in the Hayling sentence completion test, and alternating numbers/letters, in both groups (Table 4).

**Table 4:** Neuropsychological assessment

	Control (MS without pain) *n* = 16	MS with cNLP *n* = 31	Statistical test	*P* value
BICAMS battery				
CVLT-II (word list), median (IQR)	60.50 (53.25–67.25)	45.00 (41.50–58.50)	W	0.0108
CVLT-II (delayed recall), median (IQR)	15.00 (12.50–16.00)	11.00 (8.00–14.00)	W	0.0147
BVMR-R, median (IQR)	32.50 (30.00–34.25)	28.00 (22.50–31.00)	W	0.0093
SDMT, median (IQR)	62.50 (61.00–65.25)	57.00 (47.00–64.00)	W	0.1275
Executive functions: concept generation				
Fluency, letter S, mean (SD)	17.00 (4.04)	15.00 (4.94)	T	0.2243
Constrained fluency, letter T, median (IQR)	9.00 (7.00–11.75)	8.50 (7.00–11.00)	W	0.6551
Executive functions: inhibition of extraneous information				
Elevator test with distraction, median (IQR)	9.00 (8.60–10.00)	9.50 (5.75–10.00)	W	0.7086
Executive functions: cognitive flexibility				
Card sorting (1), median (IQR)	7.00 (6.00–7.00)	5.00 (5.00–6.00)	W	0.0006*
Card sorting (2), median (IQR)	5.00 (4.50–6.00)	5.00 (5.00–6.00)	W	0.8249
Recognizing card groups (1), median (IQR)	24.00 (21.00–24.00)	12.00 (8.00–20.00)	W	<0.0001*
Recognizing card groups (2), median (IQR)	20.00 (20.00–24.00)	12.00 (10.00–16.00)	W	0.0002*
Reverse digit span, median (IQR)	6.00 (6.00–7.25)	7.00 (6.00–8.00)	W	0.5979
Alternating numbers and letters), median (IQR)	12.00 (9.00–12.00)	12.00 (12.00–12.00)	W	0.08504
Hayling sentence completion, median (IQR)	11.00 (10.00–12.00)	11.00 (9.00–11.00)	W	0.1736

*Statistical significance at 5% level after Bonferroni correction for multiple comparisons (fourteen comparisons, threshold *P* = 0.0036).

BICAMS, Brief International Cognitive Assessment in MS test battery (see text for details); BVMR-R, Brief Visuospatial Memory Test–Revised T1-3; cNLP, chronic neuropathic limb pain; Card Sorting (1 and 2) = Delis Kaplan Executive Function System Card Sorting tests, groups 1 and 2 (see ‘Materials and methods’ section for details); CVLT-II, California Verbal Learning Test-II; MS, multiple sclerosis; SDMT, Symbol Digits Modality Test; T, Students *t-*test; W, Wilcoxon rank sum test.

### Structural and functional MRI imaging

#### Availability of data

Two participants were excluded from MRI imaging, because of previously undisclosed contraindication *n* = 1, and claustrophobia *n* = 1. One participant (cNLP group) completed only structural imaging, because of illness.

#### Multiple sclerosis lesion volume and distribution

**Figure 2 Mapping of multiple sclerosis lesion distribution.**
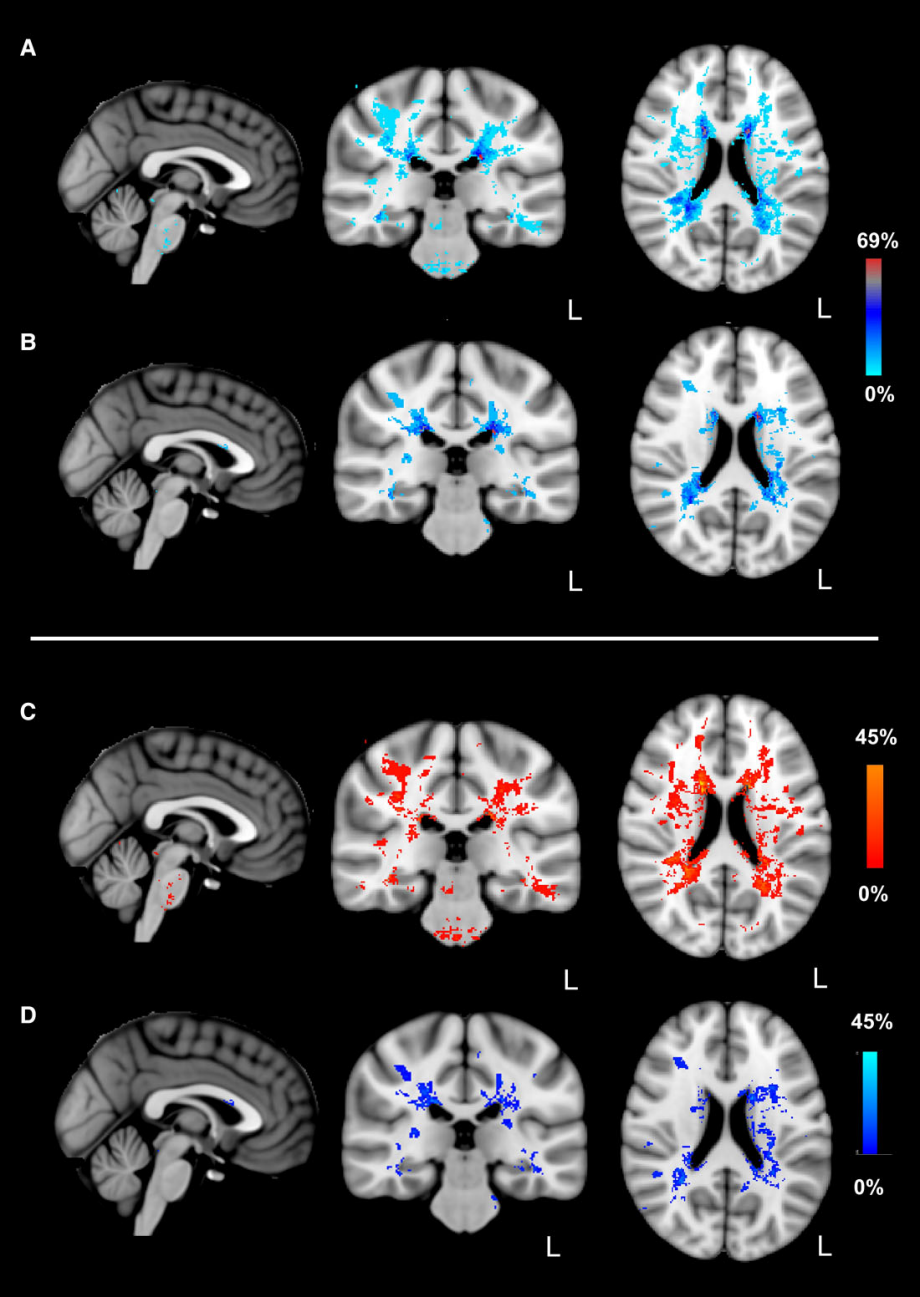
Multiple sclerosis lesion distribution probability maps. Individual binarized lesion maps were summed and averaged to create group-wise lesion probability maps for the cNLP and control groups separately. Lesion distribution in cNLP and control groups was additionally compared by subtracting lesion distribution masks with the following contrasts: (cNLP group—control group) and (control group—cNLP). (**A**) Displays lesion distribution probability in cNLP group (*n* = 29). (**B**) Displays lesion distribution probability in control group (*n* = 16). (**C**) Displays contrast produced by subtracting control group lesion distribution probability map, from cNLP group lesion distribution probability map. Contrast denotes higher probability of lesion location in cNLP than control group. (**D**) Displays contrast produced by subtracting cNLP group lesion distribution probability map from control group lesion distribution probability map. Contrast denotes higher probability of lesion location in control than cNLP group. Lesion distribution probability maps are superimposed on MNI 152 T1 1 mm brain-extracted template. Images are displayed in radiological convention; L denotes left side for coronal and axial images. Colour bar denotes probability of lesion location.

Lesion distribution maps confirmed typical distribution of multiple sclerosis lesions in both groups (Fig. 2, including subtraction map to facilitate visual comparison of group-wise lesion distribution). Total multiple sclerosis lesion volume did not differ significantly between groups. Control group median was 0.12% of ICV, IQR 0.06–0.42%; cNLP group median 0.20% of ICV, IQR 0.12–0.42%; *P* = 0.21.

#### Voxel-based morphometry

In the whole grey-matter analysis, we found no statistically significant difference in GM volume between the pain and control groups (*P* < 0.05 corrected for multiple comparisons, TFCE, 5000 iterations). Similarly, at the same statistical threshold, no statistically significant difference in GM volumes was found between groups in an analysis restricted to the described DPMS ROI. Exploratory whole-brain analyses at the statistical threshold of *P* < 0.001, uncorrected for multiple comparisons, confirmed that ROIs used for the fMRI analysis did not overlap with areas of differential GM volume, even at this exploratory threshold.

#### Functional connectivity of rostral anterior cingulate cortex seed, in pain and control groups

After exclusion of participants moving greater than 1 mm (*n* = 2), absolute head motion did not vary significantly between groups. In the cNLP group (*n* = 26) median head motion was 0.24 mm, IQR 0.16–0.30. In the control group (*n* = 16) median head motion was 0.20 mm, IQR 0.16–0.26; *P* = 0.42.

**Figure 3 Resting state functional MRI analyses.**
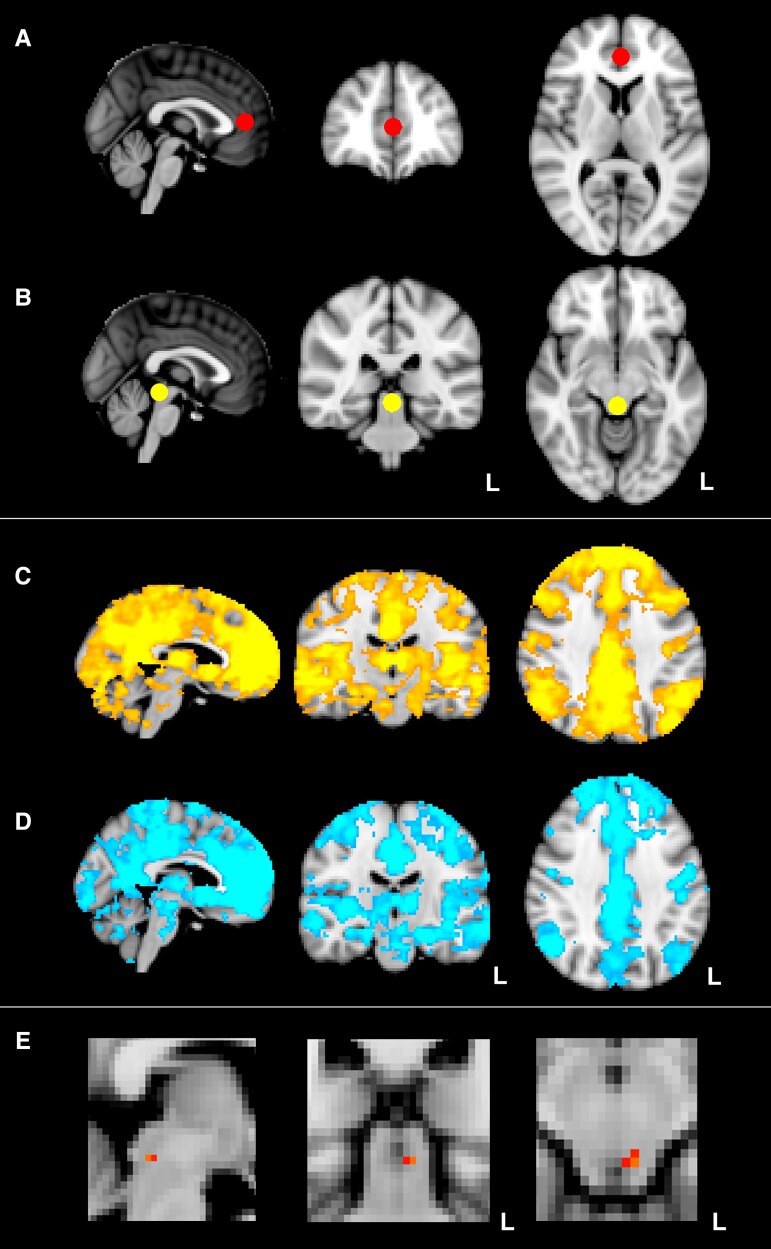
(**A** and **B**) Display region of interest masks employed in analyses. All masks were binarized spherical masks, radius 6 mm, based on existing literature. (**A**) Displays rostral ACC seed mask, centred at x = 0, y = 42, z = 8. (**B**) Displays PAG region of interest mask centred at x = 0, y = −32, z = −10. A further occipital region of interest mask (centred at x = 0, y = 74, z = 8) was used to examine specificity of functional connectivity findings to DPMS, as occipital cortex has no known role in DPMS. This occipital mask is not shown, for clarity. We then examined mean functional connectivity of the rostral ACC seed separately in the cNLP and control groups across the whole brain. (**C**) Displays mean functional connectivity of rACC seed in cNLP group. (**D**) Displays mean functional connectivity of rACC seed in control group. Functional connectivity of rACC seed with a range of cortical and subcortical structures including those involved in executive function and DPMS was identified in both groups. We then examined differential functional connectivity of the rACC seed with the specified PAG region of interest using TFCE permutation analysis. Panel (**E**) displays statistically significant higher functional connectivity of the rACC seed with PAG region of interest in control group, compared to cNLP group. Maximal differential connectivity was identified at x = −4, y = −32, z = −12; *P* < 0.05 corrected for multiple comparisons. Images are overlaid on the brain-extracted MNI 152 2 mm template, displayed in radiological convention; L denotes left side for coronal and axial images. PAG region of interest displays are cropped for clarity.

FC of the rACC seed with a range of cortical and subcortical structures, including those involved in executive function and DPMS (including frontal and prefrontal cortices, thalamus and PAG), was identified in both groups (Fig. 3).

#### Functional connectivity of rostral anterior cingulate cortex with PAG ROI

In the specified ROI analysis, FC of the rACC seed with PAG ROI was significantly higher in the control group (*P* < 0.05 after correction for multiple comparisons). Maximal differential connectivity was observed at the ventrolateral PAG (x = −4, y = −32, z = −12). Separate inclusion of brainstem-specific multiple sclerosis lesion volume, or of participant sex, as covariates of no interest in the analysis did not eliminate the observed differential connectivity, with maximal differential connectivity remaining at ventrolateral PAG (x = −4, y = −32, z = −12) in both of these analyses (both *P* < 0.05, corrected for multiple comparisons, TFCE).

FC between rACC seed and occipital cortex ‘control’ ROI did not vary to a statistically significant degree between cNLP and control groups (threshold p 0.05, corrected for multiple comparisons, TFCE, 5000 iterations) (data not shown).

#### Multiple sclerosis lesion volume affecting rostral anterior cingulate cortex:periaqueductal gray white matter tracts and brainstem

Multiple sclerosis lesion volume affecting white matter tracts between rACC and PAG was not significantly different in pain and control groups. Tract-specific lesion volume in controls was 7.4 × 10^−5^% of ICV, IQR 0.0 to 4 × 10^−4^%; in the cNLP group 7.8 × 10^−5^% of ICV, IQR 0.0 to 9 × 10^−4^%; *P* = 0.74.

Multiple sclerosis lesion volume affecting the brainstem was significantly higher in cNLP group (control group 0.0% of ICV, IQR 0.0 to 5 × 10^−4^%, cNLP group 0.0021% of ICV, IQR 0.0002–0.0047%; *P* = 0.0017).

Post-hoc permutation analysis within the described brainstem mask identified higher probability of multiple sclerosis lesion location in the cNLP group in the caudal pons. Maximal probabilities of lesion location were observed at MNI coordinates x = 0, y = −27, z = −45; x = 0, y = −26, z = −39 and x = −10, y = −24, z = −40. These coordinates were identified as pontine decussation and arcuate nuclei^54^ (all *P* < 0.05 corrected for multiple comparisons, TFCE). No brainstem lesion location probability was higher in the control group.

### Correlates of pain severity within chronic neuropathic limb pain group

#### Clinical and neuropsychological data

Pain Severity Index^27^ was significantly correlated with increased symptoms of anxiety.^34^ (Spearman’s Rho 0.58), fatigue^35^ (Rho 0.60), pain catastrophizing^37^ (Rho 0.57) and sleep quality^36^ (Rho 0.68) (all *P* < 0.001); but not depression^34^ (Rho 0.41, *P* = 0.02) (five comparisons, threshold p after Bonferroni correction 0.01).

However, neuropsychological measures were not significantly associated with pain severity index (all *P* > 0.05).

#### Structural and functional imaging data

Whole brain multiple sclerosis lesion volume, rACC:PAG tract lesion volume, brainstem-specific lesion volume, GM volumes and distribution were not significantly associated with pain severity (all *P* > 0.05). A trend to positive correlation in FC of rACC:PAG and pain severity at time of imaging was observed, but this was not statistically significant after correction for multiple comparisons in space (TFCE, *P* = 0.02, without correction for multiple comparisons in space, maximal correlation observed at x = −2, y = −28, z = −6).

## Discussion

Here, we report that presence of cNLP, a specific common pain syndrome affecting pwMS, is significantly associated with impairment in measures of mental flexibility,^8^ as well as with altered FC within the DPMS network. While we also confirm previously described associations between pain and affective disturbance in neuropathic pain conditions,^16,55^ executive dysfunction has not previously been confirmed in pwMS experiencing cNLP.^22^

We employed a disease control group of pwMS, rather than a healthy control comparator group.^19^ Use of a disease control group allowed us to account for known and unknown confounders associated with presence of multiple sclerosis specifically, and chronic disease more generally.^2^ We confirmed presence of neuropathic pain by published recommendations.^26^ To improve generalizability of our study findings to clinical populations, we did not exclude pwMS on the basis of known diagnoses of affective disorders^55^ nor on the use of analgesics (with the exception of strong opiates^29^). Sex ratio and age of our study participants are comparable to sex-specific multiple sclerosis prevalence ratios in the UK and internationally, as well as peak age of multiple sclerosis incidence for both men and women in the UK.^1,32^ Generalizability of our results to pwMS outside a UK healthcare setting, or those experiencing differing subtypes of multiple sclerosis, should be the focus of future study.

With regards to FC of key DPMS structures, participants with cNLP manifested significantly decreased FC between rACC and PAG. A whole-PAG ROI was used,^9^ in common with other groups^19^ because of a lack of prior studies highlighting specific brainstem subregions in studies of multiple sclerosis related pain.^19,56^ However, ventrolateral PAG was specifically identified within this region as the site of maximal differential FC with rACC. An important role of ventrolateral PAG in DPMS mechanisms^14,15^ (previously defined as centreing on MNI coordinates x = ±3, y = −32, z = −12^57^) has previously been identified, including altered FC with rACC in human pain conditions.^9,15^ Rostral ACC is thought to be closely involved in cognitive and emotional aspects of pain modulation including cognitive appraisal and mental flexibility, shows close structural connectivity to frontal cortex regions,^7^ and is strongly implicated in placebo analgesia mechanisms.^6, 9^

Executive dysfunction in association with multiple sclerosis-related neuropathic pain has not previously been confirmed.^22^ Our finding that reduced mental flexibility is associated with cNLP is consistent with some, but not all, studies of clinical pain syndromes.^58,59^ Our data suggest a specific deficit in mental flexibility, rather than a more general deficit in executive function, memory or processing speed^8,40^ (Table 4). An association between pain and impaired mental flexibility in pwMS could be explained by altered appraisal or re-appraisal of nociceptive inputs.^6^ Alternatively, the experience of chronic pain or associated conditions may adversely impact upon cognitive performance, although a differential effect on executive functions would be incompletely explained. An intrinsic role of PAG in cognitive processing and attentional analgesia has previously been suggested.^10^ Both brainstem and cortical mechanisms might contribute to cognitive impairments associated with chronic pain disorders.

With regards to the potential structural substrate of the described results, cNLP was not significantly associated with volume of key DPMS-relevant GM brain structures, nor with overall volume of multiple sclerosis brain lesions, or volume of lesions specifically affecting rACC:PAG tracts. We acknowledge that such influences are not ruled out, however, and larger future studies could usefully confirm these results.

The described higher volume of multiple sclerosis lesions affecting the brainstem in the cNLP group has not previously been identified in cNLP, yet is consistent with the previously identified role of brainstem-mediated DPMS mechanisms in clinical chronic pain states.^5,6,13–15^ However, any mechanism by which brainstem lesions might exert an effect on DPMS mechanisms should be both confirmed, and further explored, in future studies. Disruption of descending pathways from PAG to RVM and the spinal cord, or of ascending nociceptive pathways, could contribute to imbalance in facilitatory and inhibitory pain modulation. The differential location of brainstem lesions described, particularly affecting the caudal pons, should be interpreted with caution. However, a close anatomical association with the rostral ventromedial medulla could be compatible with impairment in PAG connectivity to this key DPMS node. Simultaneous spinal and supraspinal fMRI imaging could contribute to resolving in future any contribution to modulation of nociceptive afferents at the spinal level.

Regarding potential confounding factors, gabapentin and pregabalin were more frequently used in the cNLP group. While use of analgesics in our study population could improve generalizability to clinical populations, these data should be interpreted with care. We do not report analyses restricted to the subgroup of participants not receiving adjuvant analgesics, because of small numbers and reduced statistical power. However, there is little clinical evidence, including at literature review^60^ to suggest that these medications might impair mental flexibility specifically or neuropsychological function more generally. In adults with peripheral neuropathic pain no association of anticonvulsant or antidepressant analgesics with impaired executive functions^59^ was found. Therefore, existing evidence does not confirm a role of gabapentinoid medications in our clinical and neuropsychological findings, though any such associations are difficult to exclude and could be a focus of future investigation. While an influence of gabapentin on stimulus-related deactivations in a human central sensitization model^61^ might reflect alterations in resting state network connectivity, any effect on rACC:PAG resting state connectivity is not established in existing literature.

Reports of affective symptoms and fatigue were significantly higher in the group with cNLP. We considered whether these factors represent a confounding influence. Depression and anxiety are frequently associated with prevalence and incidence of pain disorders^16^ and a close theoretical relationship between cognitive and affective measures and pain is described.^6,58^ Similarly, fatigue in our view is closely linked to chronic pain experience, rather than representing a confounder. Fatigue and depression might be hypothesized to affect neuropsychological performance, and have previously been linked to impaired processing speed and deficits in fluency and recall in pwMS.^62^ However, the pattern of neuropsychological performance reported in the current study has not previously been reported in association with depression and fatigue in multiple sclerosis. We note also that, while anxiety and fatigue were significantly correlated with pain severity, cognitive measures were not. These findings suggest that the reported neuropsychological variables are unlikely to simply reflect sequelae of affective disturbance or fatigue. We note that our available sample size did not allow multivariate modelling of the clinical and neuropsychological associations of pain severity, within the group experiencing cNLP.

The potential role of technical factors in the reported fMRI results is a possible limitation, and is carefully considered. Firstly, GM volumes in the fMRI ROIs analysed in this study did not vary significantly between groups in a VBM analysis, even in an additional exploratory analysis employing a less stringent statistical threshold. Secondly, shared CSF signal between regions is known to either reduce or increase apparent FC, depending on CSF dynamics.^9,14^ To mitigate against this risk, manual denoizing of rs-fMRI data (using Independent Component Analysis) was employed. Subsequently, inclusion of a CSF timecourse as a nuisance covariate in the analysis was used. Next, inspection of mean rs-fMRI FC of the rACC seed (Fig. 3) additionally confirmed no significant correlation with time courses of CSF voxels. Furthermore, a ‘region of no interest’ analysis (examining FC of rACC with a ROI in occipital cortex, which has no known role in DPMS but is placed in the midline similarly to the rACC and PAG regions of interest described) demonstrated no significant correlation between rACC and occipital time courses. Thirdly, participant head motion is known to induce spurious correlations in resting BOLD time courses due to shared variance.^63^ Therefore we used a stringent threshold^14^ to exclude participants moving more than 1 mm during rs-fMRI acquisition (before ICA data denoizing). After exclusion of these participants, average head movement did not vary significantly between groups. Head motion parameters were also included in the described analyses. Use of 3 mm slice thickness and a FWHM of 5 mm in our fMRI acquisition may have limited our ability to disambiguate fMRI activation patterns between small brainstem nuclei; nonetheless, it afforded good SNR and is consistent with existing literature, in terms of slice thickness^13,14,19^ and spatial smoothing.^13^

In conclusion, focussed clinical, behavioural and imaging assessments suggest that cognitive and affective dysfunction are associated with reduced FC of DPMS nodes, in cNLP associated with multiple sclerosis. These findings, in a small study, support previous studies suggesting a key role of the DPMS in human chronic pain states, yet add to limited understanding of the role of cognitive dysfunction in particular.^14,15^ The finding of diminished rACC: ventrolateral PAG FC in the cNLP group could suggest disrupted descending pain inhibition in cNLP. No difference in whole-brain lesion volume was confirmed. However, the role of brainstem lesions in particular should be further confirmed and explored, with emphasis on disruption to specific DPMS pathways. These data add to growing understanding of dysfunction in endogenous pain modulatory systems associated with chronic neuropathic pain in the injured CNS.

## Supplementary Material

fcac124_Supplementary_Data

## Abbreviations

BICAMS =: Brief International Cognitive Assessment for Multiple Sclerosis
BVMR-R =: Brief Visuospatial Memory Test-Revised
CVLT-II =: California Verbal Learning Test-II
cNLP =: chronic neuropathic limb pain
DKEFS =: Delis Kaplan Executive Function System
DPMS =: descending pain modulatory system
DMT =: disease modifying therapy for multiple sclerosis
DLPFC =: dorsolateral prefrontal cortex
EDSS =: Expanded Disability Status Scale
FSS =: Fatigue Severity Scale
FLAIR =: Fluid-Attenuated Inversion Recovery
FEAT =: FMRIB Expert Analysis Tool
FLAME =: FMRIB Local Analysis of Mixed Effects
FSL =: FMRIB Software Library
FWHM =: full width half maximum
FC =: functional connectivity
GM =: grey matter
HADS =: Hospital Anxiety and Depression Scale
ICA =: independent components analysis
ICV =: intracranial volume
MRI/fMRI/rs-fMRI =: magnetic resonance imaging/functional magnetic resonance imaging/resting state functional magnetic resonance imaging
MNI =: Montreal Neurosciences Institute
MELODIC =: Multivariate Exploratory Linear Optimized Decomposition into Independent Components
PAG =: periaqueductal gray
PSQI =: Pittsburgh Sleep Quality Inventory
QST =: quantitative sensory testing
ROI =: region of interest
rACC =: rostral anterior cingulate cortex
SPM =: Statistical Parametric Mapping
SDMT =: Symbol Digit Modality Test
TFCE =: threshold free cluster extent
VBM =: voxel based morphometry
WUR =: wind up ratio.
