# Supplementary material for: Coupling cognitive and brainstem dysfunction in multiple sclerosis-related chronic neuropathic limb pain
Source: Brain Commun. 2022 May 17;4(3):fcac124. doi: 10.1093/braincomms/fcac124 (PMC9155950; doi:10.1093/braincomms/fcac124)
Supplement: fcac124_Supplementary_Data [file fcac124_supplementary_data.docx]

Supplement:

Foley et al, Coupling cognitive and brainstem dysfunction in multiple sclerosis-related chronic neuropathic limb pain

| **Contents**: |
| --- |
| Supplementary Table 1:  Individual Participant Medication;  MS chronic neuropathic limb pain group and MS control group |

| Supplementary Table 1: Individual Participant Medication | | | | | | | | | | | |
| --- | --- | --- | --- | --- | --- | --- | --- | --- | --- | --- | --- |
| Subject number | Age band  (years) | Non-analgesic medication | |  | Adjuvant Analgesics | | | | Paracetamol/  NSAID/  weak opiate | MS Disease Modifying Therapy | Baclofen |
|  |  |  | | Antidepressant  with no established analgesic role (eg SSRI) | Tricyclic | GBP/PGB | SNRI | CBZ |  |  |  |
|  |  | **STUDY GROUP:**  **MS CHRONIC NEUROPATHIC LIMB PAIN** | |  |  |  |  |  |  |  |  |
| 1 | 40-44 |  | |  | AMI 50mg nocte |  |  |  |  |  |  |
| 2 | 45-49 |  | |  |  |  |  |  | Paracetamol PRN, Ibuprofen PRN | Beta-Interferon |  |
| 3 | 40-44 | none | |  |  |  |  |  |  |  |  |
| 4 | 50-54 | Flavoxate 1tab od, Fluoxetine 40mg od | | Y |  | PGB 300mg bd |  |  |  | Fingolimod | Baclofen 60mg/24h |
| 5 | 60-64 | Amantadine, Tamsulosin, Clonazepam | |  | AMI 40mg nocte | GBP 1800mg/24hrs |  |  | Ibuprofen (occasional) | Beta interferon |  |
| 6 | 20-24 | Oral Contraceptive Pill | |  |  | GBP 300mg nocte |  |  |  | Natalizumab |  |
| 7 | 35-39 | Vitamin D, Multivitamin | |  |  |  |  |  |  | Glatiramer  Acetate |  |
| 8 | 50-54 | Lansoprazole, Simvastatin, Lisinopril, Folic Acid, Thiamine, Sertraline | | Y |  | PGB 200mg/24h |  |  |  | Beta-interferon | Baclofen 40mg/24h |
| 9 | 30-34 | Amantadine, Fluoxetine 20mg od, Oral Contraceptive Pill, Erythromycin, Thyroxine | | Y | Nortriptyline 10mg nocte | GBP 1200mg/24h |  |  | Tramadol 100mg od | Glatiramer Acetate |  |
| 10 | 35-39 |  | |  | AMI 30mg nocte | GBP 1800mg/24h |  |  | Dihydrocodeine 30mg PRN | Fingolimod |  |
| 11 | 60-64 | Nasal Decongestant | |  | AMI 10-20mg nocte |  |  |  |  |  |  |
| 12 | 50-54 | Salbutamol, Venlafaxine 75mg od | |  |  |  |  |  |  | Dimethyl Fumarate |  |
| 13 | 40-44 | Atenolol, Oral Contraceptive, Vitamin D, Sumatriptan (not received for several weeks) | |  | AMI 20mg nocte | GBP 900mg/24h |  |  |  | Beta-Interferon |  |
| 14 | 40-44 |  | |  |  |  |  |  | Co-codamol 30/500 PRN, approx. 180mg codeine/24h |  |  |
| 15 | 45-49 | Cetirizine, Lansoprazole, Bendroflumethiazide, Amlodipine, Thyroxine, | |  |  | PGB 300mg/24h |  |  | Co-codamol, strength unknown, PRN. Ibuprofen 200mg prn | Glatiramer Acetate |  |
| 16 | 45-49 | Ventolin, Seretide | |  |  |  |  |  |  |  |  |
| 17 | 55-59 |  | |  | AMI 10mg nocte |  |  |  | Paracetamol 2-3 grams/24h, Aspirin + caffeine (2 per week) |  |  |
| 18 | 40-44 | Eye drops (steroid, cyclopentolate), Antihistamines, Vitamin D, Vitamin C | |  | AMI 50mg/nocte | GBP 900mg/24h |  |  |  |  |  |
| 19 | 45-49 | Fluoxetine 30mg, Lactulose, Hyoscine, Omeprazole, Loperamide, Methylcellulose | | Y |  | GBP 600-900mg/24h |  |  | Paracetamol approx. 2g/24h PRN , Etoricoxib 60mg PRN, approx 3/week |  | Baclofen 10mg/24h |
| 20 | 35-39 | Ferrous Sulphate | |  |  |  |  |  | Ibuprofen 600mg/week |  |  |
| 21 | 35-39 | Sertraline, Methylcellulose, Oral Contraceptive Pill | | Y |  | GBP 2700mg/24h |  |  |  | Glatiramer Acetate |  |
| 22 | 35-39 |  | |  |  | PGB 200mg/24h | Duloxetine 60mg |  | Paracetamol PRN with interferon | Beta-interferon |  |
| 23 | 45-49 | Oral Contraceptive Pill, oral acyclovir | |  |  |  |  |  |  | Beta-interferon |  |
| 24 | 35-39 |  | |  |  |  | Duloxetine ?60mg |  | Co-codamol 30/500 up to qds (PRN) | Fingolimod |  |
| 25 | 40-44 | Stemetil, Diazepam 2mg prn | |  | AMI 25mg nocte | GBP 2500mg/24h |  |  | Co-codamol 30/500 (PRN) |  |  |
| 26 | 60-64 | Alendronate, Evening Primrose Oil, Vitamin D, anti-oxidant supplement, cranberry supplement | |  |  |  |  |  | Paracetamol and Ibuprofen PRN infrequently with interferon | Beta-interferon |  |
| 27 | 30-34 | Citalopram | | Y | AMI 75mg nocte | Pregabalin 300mg/24h |  |  |  | Fingolimod |  |
| 28 | 50-54 | Lisinopril, Bendroflumethiazide | |  |  | PGB 450mg/24h |  |  | Paracetamol PRN occasional |  |  |
| 29 | 25-29 | Omeprazole, Gaviscon, | |  | AMI 30mg nocte | PGB ?300mg/24h |  |  | Co-codamol 8/500 1 tab infrequently. Ibuprofen infrequently | Beta-interferon | Baclofen dose unclear |
| 30 | 55-59 | Simvastatin 40mg, Candesartan 4mg | |  |  | GBP PRN |  |  |  |  |  |
| 31 | 35-39 | Inhalers, Oral Contraceptive Pill, Vitamin D | |  |  |  |  |  |  | Beta-interferon |  |
|  |  | **STUDY GROUP:**  **MS CONTROLS** | |  |  |  |  |  |  |  |  |
| 1 | 45-49 |  | |  | AMI 75mg nocte |  |  |  |  | Dimethyl Fumarate |  |
| 2 | 40-44 | Hydroxycobalamin, Omeprazole, Vitamin D, | |  | AMI 20mg nocte |  |  |  | Aspirin (concurrently with DMF) | Dimethyl Fumarate |  |
| 3 | 25-29 | Solifenacin, Ventolin, Sildenafil PRN | |  |  |  |  |  |  | Dimethyl Fumarate |  |
| 4 | 40-44 | Oral contraceptive pill | |  |  |  |  | Tegretol Retard 400mg bd | Paracetamol PRN | Natalizumab |  |
| 5 | 35-39 | Vitamin D, Hydroxycobalamin | |  |  |  |  |  |  | Dimethyl Fumarate |  |
| 6 | 50-54 | Fluoxetine 20mg od, melatonin | | Y |  |  |  |  |  | Beta-interferon |  |
| 7 | 30-34 | Oral Contraceptive Pill | |  |  |  |  |  |  |  |  |
| 8 | 30-34 | Mirtazapine 45mg nocte, longterm Amoxycillin, Multivitamins, Oxybutynin, | | Y | AMI 100mg nocte |  |  |  |  | Beta-interferon | Baclofen 30mg/24h |
| 9 | 55-59 | Thryoxine, Fexofenadine, Adcal D3, Citalopram, Vesicare | | Y |  |  |  |  |  |  |  |
| 10 | 50-54 |  | |  |  |  |  |  |  | Glatiramer Acetate |  |
| 11 | 55-59 | Loratadine, nasal decongestant spray | |  |  |  |  |  |  | Fingolimod |  |
| 12 | 25-29 | Vitamin D, antihistamine | |  |  |  |  |  |  | Dimethyl Fumarate |  |
| 13 | 40-44 | Venlafaxine 375mg/24h | | Y |  |  |  |  |  | Beta-interferon |  |
| 14 | 30-34 |  | |  |  |  |  |  |  | Dimethyl Fumarate |  |
| 15 | 25-29 | Vitamin D | |  |  |  |  |  |  | Dimethyl Fumarate |  |
| 16 | 50-54 | Aspirin 75mg od | |  |  |  |  |  | Occasional Ibuprofen, occasional Paracetamol | Dimethyl Fumarate |  |
|  | | | NSAID = Non-Steroidal Anti-Inflammatory Drug  PRN = as required (pro re nata)  GBP = Gabapentin  PGB = Pregabalin  SNRI = Serotonin and Noradrenaline Reuptake Inhibitor  CBZ = Carbamazepine  AMI = Amitriptyline  mg = milligram  24h = 24 hours  ? = exact dose unclear  Study number is allocated for the purpose of display and is not the same as subject’s anonymous study ID number used for analysis  Medication doses are given where available  Where name of medication was not available (for instance purchased by patient), class of drug is indicated | | | | | | | | |
